# Association of plasma homocysteine with the atherogenic index of plasma and modification by body mass index: a cross-sectional study

**DOI:** 10.3389/fcvm.2026.1806051

**Published:** 2026-07-15

**Authors:** Bin Jiang, Juan Yu, Chongxiang Chen, Yongshi Huang

**Affiliations:** Department of Health Management, Guangzhou Development District Hospital, Guangzhou, Guangdong Province, China

**Keywords:** atherogenic index of plasma, body mass index, cross-sectional study, homocysteine, mediation analysis, obesity

## Abstract

**Objective:**

Hyperhomocysteinemia is generally considered an independent risk factor for cardiovascular disease; nevertheless, the independent connection between plasma homocysteine (HCY) and the atherogenic index of plasma (AIP), which integrates the risk of atherogenic lipids, has not been completely elucidated. The potential biological mechanisms relating HCY to AIP also need to be clarified further. This investigation investigates the relation between plasma HCY and AIP in a general adult group, examines whether inflammation and liver function statistically influence this relation, and evaluates the effect of body mass index (BMI) on the relationship. In this cross-sectional study, 887 adults were analyzed in the final stage. Outliers were excluded based on a lenient threefold interquartile range criterion to maintain biologically reasonable variations. The relationship between HCY and continuous AIP was analyzed by multiple linear regression, while the relation with high AIP risk was studied by logistic regression. Mediation analysis was performed using the bootstrap method, with white blood cell count (WBC) and alanine aminotransferase (ALT) as putative mediators. Subgroup analysis and formal interaction tests were conducted to investigate if the association between HCY and AIP varied among different clinical groups.

**Results:**

Plasma HCY was positively correlated with AIP (*r* = 0.294, *P* < 0.001). In multivariable linear regression, each 1 μmol/L increase in HCY was associated with a 0.0113-unit increase in AIP after adjustment for potential confounders (*P* < 0.001). Logistic regression showed that each 1 μmol/L increase in HCY was associated with a higher likelihood of high AIP risk (OR = 1.282, 95% CI: 1.186–1.387). Bootstrap mediation analyses did not identify significant indirect effects through either WBC or ALT. Subgroup analyses further showed that the positive association between HCY and AIP was stronger in participants with BMI ≥ 24 kg/m (=0.014) than in those with BMI < 24 kg/m (=0.008), with significant effect modification by BMI (*P* for interaction = 0.006).

**Conclusion:**

Plasma HCY was independently and positively associated with AIP, and this relationship was more pronounced among overweight or obese participants. The association appeared to be driven mainly by a direct pathway, with no statistically significant mediation through the selected markers of inflammation or liver function. These findings suggest that BMI should be considered when evaluating the lipid-related atherogenic risk associated with elevated HCY.

## 1 Introduction

Atherosclerotic cardiovascular disease (ASCVD) is still one of the main causes of death and disability all over the world (1, 2). In addition to the conventional risk factors such as hypertension, diabetes and smoking, homocysteine (HCY), an amino acid with sulfur which is formed during the metabolism of methionine, has been observed to be connected with ASCVD risk in several research papers (3–6). Though there is enough evidence for this connection, the precise mechanisms by which HCY affects atherosclerosis have not been clearly explained yet. It is indicated that this association may be related to endothelial damage, oxidative stress, inflammatory reactions and changes in lipid metabolism (7).

The atherogenic index of plasma (AIP), defined as the base-10 logarithm of the triglyceride to high-density lipoprotein cholesterol ratio, is a useful comprehensive indicator for evaluating the atherogenicity of plasma (8). In comparison with single lipid measurements, AIP gives a more integrated reflection of the metabolism of triglyceride-rich lipoproteins and is closely related to the existence of small, dense low-density lipoprotein particles, which possess particularly strong atherogenic effects (9, 10). However, the independent association between HCY and AIP in the whole population has not been studied in detail yet.

The effects of HCY on AIP are not yet clear. It is possible that inflammation and liver function may be the underlying biological mechanisms. HCY can lead to an increase of monocytes and an enhanced secretion of proinflammatory cytokines; furthermore, systemic inflammation may worsen the atherosclerosis and lipid metabolic disorder (11). The liver is involved in both the metabolism of HCY and the synthesis of lipoproteins; hence, hepatic disorder may affect the clearance of HCY and change the lipoprotein concentrations in circulation (12). However, the influence of the inflammatory condition or liver function on the connection between HCY and AIP still needs to be studied by further research.

The influence of HCY may differ among different metabolic groups. Obesity, usually calculated by body mass index (BMI), is an important factor affecting cardiometabolic disorders and can intensify the vascular damage related to hyperhomocysteinemia via insulin resistance, persistent low-level inflammation and abnormal adipose tissue function. It is clinically significant to investigate whether BMI influences the relationship between HCY and AIP in order to identify those individuals with a higher atherogenic risk.

According to this, a cross-sectional analysis was performed on an adult health examination group with three purposes: (1) to investigate the independent relationship between plasma HCY and AIP; (2) to investigate whether the white blood cell count (WBC), indicating systemic inflammation, and alanine aminotransferase (ALT), indicating liver function, mediate this relationship; and (3) to study the consistency of the relationship in different subgroups classified by sex, age, body mass index and hypertension status, paying special attention to the effect modification by body mass index.

## 2 Methods

### 2.1 Study design and population

This cross-sectional study used data from the 2024 health examination database of Guangzhou Development District Hospital. The hospital provides routine health examination services for employees from several enterprises and institutions. During the study period, 937 consecutive adults who completed health examinations were screened initially. All participants were aged 18 years or older. Because the database did not systematically collect occupational category, educational attainment, or household income, detailed adjustment for socioeconomic status was not possible. Written informed consent was obtained from all participants, and the study protocol was approved by the Medical Ethics Review Committee of Guangzhou Development District Hospital (Approval No.: F2026-004).

### 2.2 The exclusion criteria were as follows

**Figure 1:**
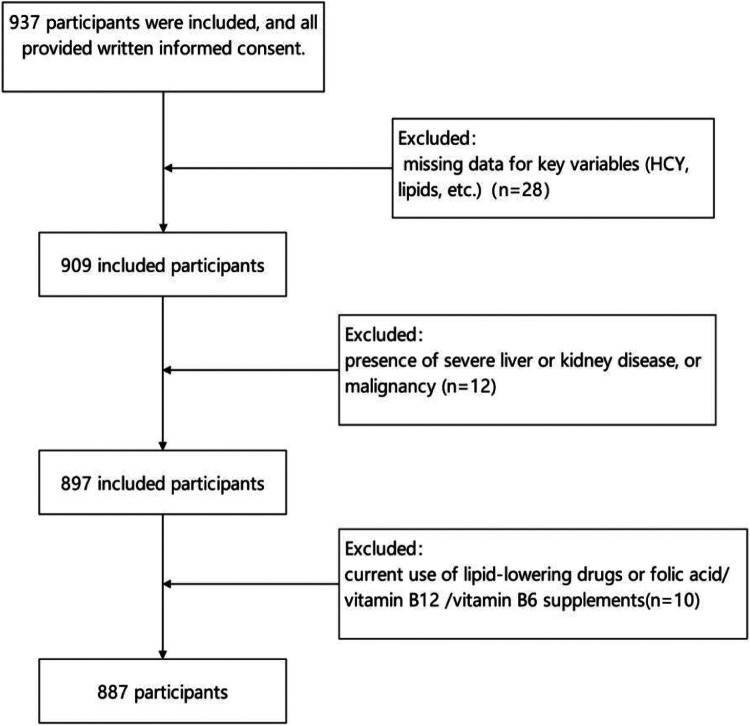
Participant flow diagram.

(1) missing values for key variables, including HCY, the lipid profile, or any prespecified covariates; (2) severe liver disease, severe kidney disease, or malignant tumor; and (3) current use of lipid-lowering medications or supplements known to influence HCY metabolism, including folic acid, vitamin B6, and vitamin B12. The participant screening process is shown in Figure 1.

### 2.3 Study variables and measurements

AIP was calculated using the following formula: AIP = log10[triglycerides [mmol/L]/high-density lipoprotein cholesterol [mmol/L]]. In the present study, high-risk AIP was defined as an AIP value in the highest quartile of the sample distribution. The 75th percentile of AIP was 0.20. Although AIP > 0.24 has generally been recommended as a threshold for high cardiovascular risk in previous literature (13), no unified cut-off value has been established for the Chinese population. We therefore used the upper quartile of the study distribution to maintain balanced group sizes and to support stable regression estimates.

WBC was selected as a candidate inflammatory mediator and was measured using an automated hematology analyzer. On the day of the physical examination, participants were routinely asked whether they had experienced fever, colds, or other acute infectious diseases during the preceding two weeks. Individuals reporting such conditions were advised to postpone the examination until recovery; therefore, participants included in the analysis had no confirmed acute infection at the time of assessment. ALT, selected as a candidate marker of liver function, was measured using the rate method. Covariates included age, sex, BMI, systolic blood pressure (SBP), fasting blood glucose (FBG), smoking status (yes/no), alcohol consumption (yes/no), history of hypertension (yes/no), history of diabetes (yes/no), history of hyperlipidemia (yes/no), exercise frequency (none, 1–2 times per week, or ≥3 times per week), and self-rated sleep quality (poor, fair, or good).

### 2.4 Statistical analysis

All statistical analyses were performed using SPSS Statistics software (version 26.0; IBM Corp.), and mediation analyses were conducted with the SPSS PROCESS macro. Before the main analyses, the key continuous variables, HCY and AIP, were assessed for extreme values. To maximize sample retention while removing implausible outliers, we applied a lenient 3× interquartile range (3 × IQR) criterion. Values below Q1 − 3 × IQR or above Q3 + 3 × IQR were considered extreme outliers and excluded. This strategy was chosen because moderate variation in HCY and AIP is biologically plausible, whereas overly stringent trimming could remove valid clinical information. The final sample size was considered sufficient for the planned multivariable regression analyses. No outlier removal was performed for other continuous variables, including BMI, SBP, FBG, WBC, and ALT. Participants were then categorized into quartiles according to plasma HCY levels.

Multiple linear regression, with AIP as the dependent variable, and logistic regression, with high AIP risk as the dependent variable, were used to examine the association with HCY. Three nested models were constructed. Model 1 was unadjusted. Model 2 was adjusted for age and sex. Model 3 was further adjusted for BMI, SBP, FBG, smoking status, alcohol consumption, history of hypertension, history of diabetes, and history of hyperlipidemia. Bootstrap mediation analysis with 5,000 resamples was used to decompose the HCY-AIP association into indirect pathways through WBC and ALT and a remaining direct pathway. Because the cross-sectional design cannot establish the temporal order among exposure, mediator, and outcome, the mediation analysis should be interpreted as a statistical decomposition of associations rather than as evidence of causal mediation. Finally, subgroup analyses and interaction tests were performed according to sex, age (<50 or ≥50 years), BMI (<24 or ≥24 kg/m), and hypertension status. All statistical tests were two-sided, and P < 0.05 was considered statistically significant.

## 3 Results

### 3.1 Baseline characteristics of the study population

Of the 937 individuals initially screened, 50 were excluded because of missing key variables, relevant medication or supplement use, serious underlying disease, or extreme HCY or AIP values beyond the prespecified 3 × IQR range. The final analytic cohort therefore included 887 participants. Baseline characteristics according to HCY quartile are summarized in Table 1. Across increasing HCY quartiles, age, BMI, SBP, AIP, and WBC showed significant upward trends (all *P* for trend < 0.05). The proportions of men, current smokers, and current alcohol consumers were also higher in the highest HCY quartile (all *P* < 0.01). By contrast, FBG, history of hypertension, diabetes, hyperlipidemia, exercise frequency, and self-rated sleep quality did not show significant trends across HCY categories.

**Table 1:** Baseline characteristics of participants according to HCY quartile.

Variable	All (*n* = 887)	Q1 (*n* = 224)	Q2 (*n* = 216)	Q3 (*n* = 229)	Q4 (*n* = 218)	*P*-value	*P* for trend
Age (years)	43.00[29.00–51.00]	39.00[29.00–50.00]	42.00[30.00–50.00]	43.00[30.00–52.00]	47.00[31.25–52.00]	0.007	<0.001
BMI (kg/m^2^)	23.99[21.99–25.83]	23.52[21.02–25.43]	23.84[22.00–25.74]	24.08[22.16–25.93]	24.46[22.41–26.16]	0.004	<0.001
SBP (mmHg)	121.00[112.00–130.00]	117.00[108.75–126.00]	121.00[112.00–131.00]	121.00[113.00–131.00]	124.00[113.00–132.00]	<0.001	<0.001
FBG (mmol/L)	4.61[4.35–4.93]	4.61[4.35–4.97]	4.58[4.37–4.86]	4.58[4.32–4.88]	4.64[4.36–4.96]	0.555	0.705
HCY (*μ*mol/L)	9.40[8.10–11.00]	7.30[6.67–7.80]	8.80[8.50–9.10]	10.10[9.70–10.70]	12.20[11.50–13.30]	<0.001	<0.001
AIP	0.12[0.06–0.20]	0.09[0.03–0.15]	0.12[0.06–0.17]	0.13[0.06–0.21]	0.16[0.10–0.24]	<0.001	<0.001
WBC (×10⁹/L)	6.81[5.87–7.81]	6.39[5.74–7.53]	7.02[5.99–8.02]	6.77[5.99–7.82]	6.89[5.93–7.83]	0.028	0.02
ALT (U/L)	20.00[14.00–30.00]	18.00[12.00–30.25]	22.00[15.00–31.00]	19.00[14.00–30.00]	21.00[15.00–28.75]	0.045	0.284
Male, *n*(%)	802 (90.4%)	160 (71.4%)	204 (94.4%)	221 (96.5%)	217 (99.5%)	<0.001	
Smoker, *n*(%)	276 (31.1%)	48 (21.4%)	68 (31.5%)	77 (33.6%)	83 (38.1%)	0.001	
Alcohol consumer, *n*(%)	212 (23.9%)	35 (15.6%)	52 (24.1%)	57 (24.9%)	68 (31.2%)	0.002	
Hypertension, *n*(%)	111 (12.5%)	24 (10.7%)	23 (10.6%)	29 (12.7%)	35 (16.1%)	0.278	
Diabetes, *n*(%)	45 (5.1%)	15 (6.7%)	11 (5.1%)	8 (3.5%)	11 (5.0%)	0.491	
Hyperlipidemia, *n*(%)	128 (14.4%)	28 (12.5%)	22 (10.2%)	39 (17.0%)	39 (17.9%)	0.066	
Exercise Frequency, *n*(%)						0.829	
None	234 (26.4%)	62 (27.7%)	57 (26.4%)	53 (23.1%)	62 (28.4%)		
1–2 times/week	483 (54.5%)	120 (53.6%)	121 (56.0%)	126 (55.0%)	116 (53.2%)		
≥3 times/week	170 (19.2%)	42 (18.8%)	38 (17.6%)	50 (21.8%)	40 (18.3%)		
Sleep Quality, *n*(%)						0.181	0.31
Poor	135 (15.2%)	26 (11.6%)	39 (18.1%)	43 (18.8%)	27 (12.4%)		
Fair	545 (61.4%)	140 (62.5%)	124 (57.4%)	138 (60.3%)	143 (65.6%)		
Good	207 (23.3%)	58 (25.9%)	53 (24.5%)	48 (21.0%)	48 (22.0%)		

HCY, homocysteine; AIP, atherogenic index of plasma; WBC, white blood cell count; ALT, alanine aminotransferase; BMI, body mass index; SBP, systolic blood pressure; FBG, fasting blood glucose. Values are shown as median [interquartile range] or number of participants (%).

### 3.2 Association between plasma homocysteine and the atherogenic index of plasma

**Figure 2:**
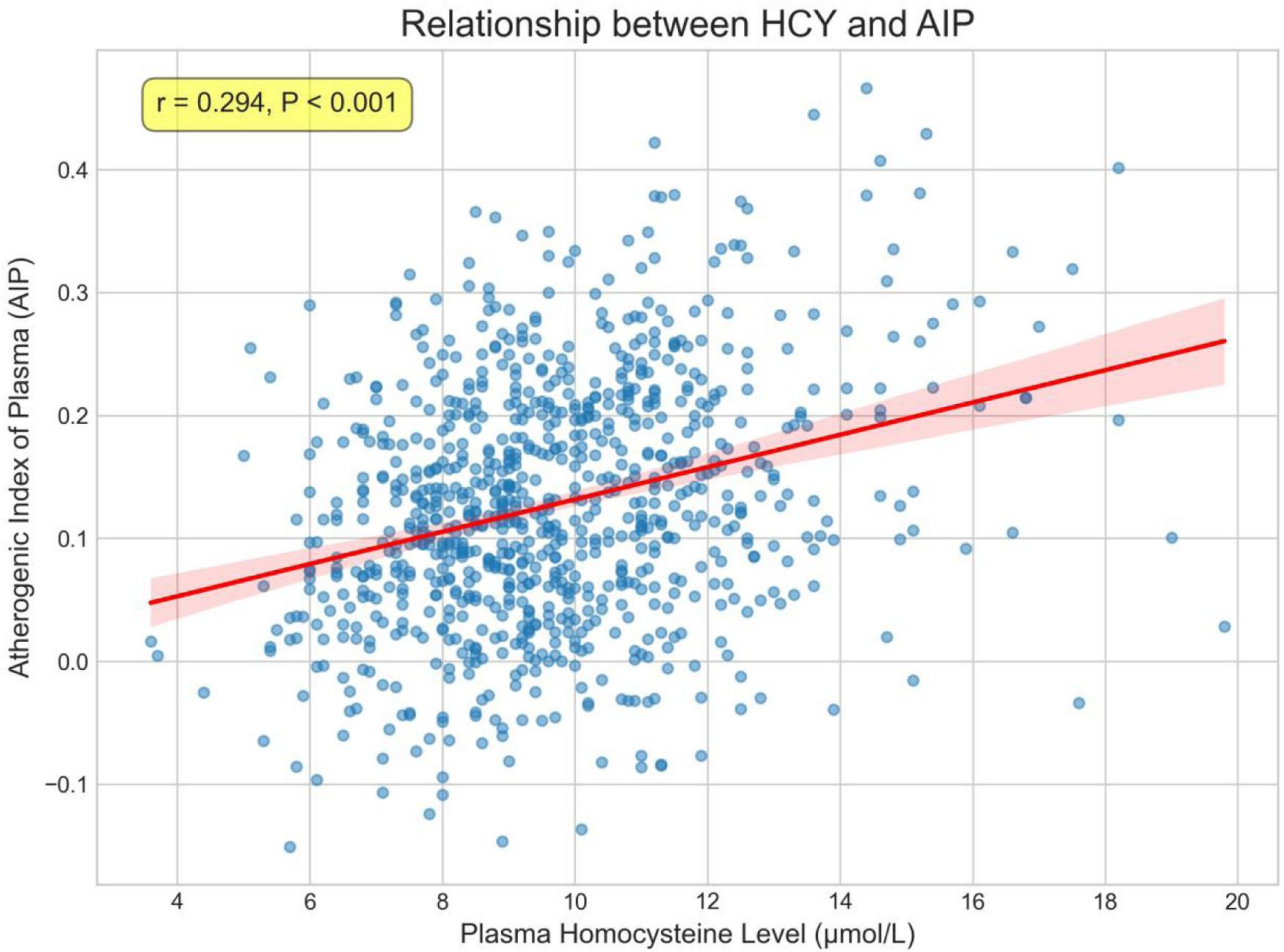
Scatter plot showing the relationship between plasma HCY and AIP. HCY, homocysteine; AIP, atherogenic index of plasma.

**Figure 3:**
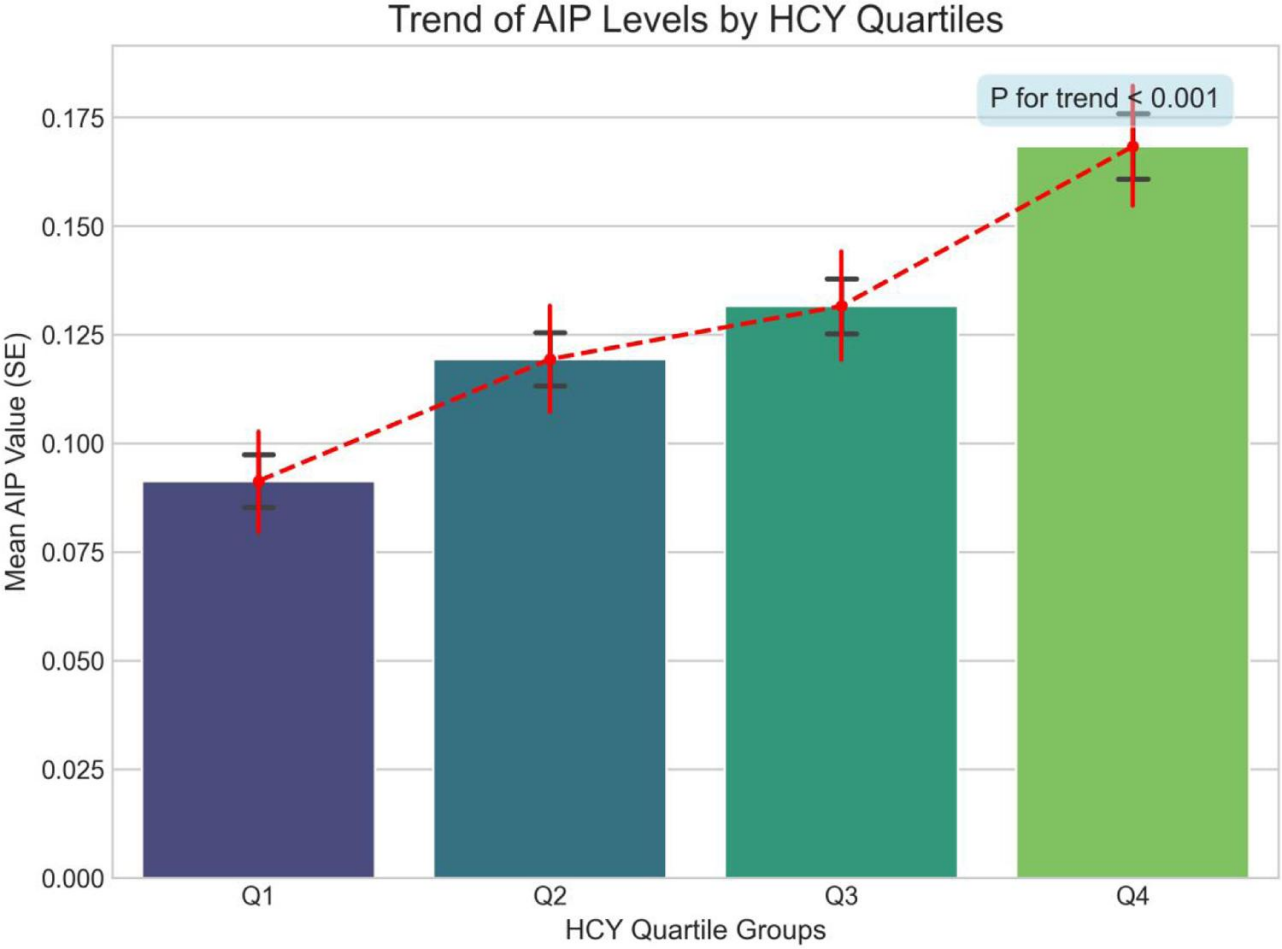
Comparison of AIP levels across plasma HCY quartiles. HCY, homocysteine; AIP, atherogenic index of plasma.

The relationship between HCY and AIP was first examined graphically using a scatter plot (Figure 2), which showed a statistically significant positive linear correlation between the two variables (Pearson *r* = 0.294, *P* < 0.001). In addition, the mean AIP value increased in a graded pattern across ascending HCY quartiles (Figure 3; *P* for trend < 0.001), supporting a dose-response association.

We then used multivariable regression to quantify this association (Table 2). In the unadjusted model (Model 1), HCY was positively associated with AIP (beta = 0.0131, *P* < 0.001). The association remained evident after adjustment for age and sex (Model 2, beta = 0.0124, *P* < 0.001) and persisted after additional adjustment for BMI, SBP, FBG, smoking status, alcohol consumption, and histories of hypertension, diabetes, and hyperlipidemia (Model 3, beta = 0.0113, *P* < 0.001). Logistic regression produced consistent results when high AIP risk, defined as the highest quartile of AIP, was used as the outcome (Table 3). In the fully adjusted model, each 1 micromol/L increase in HCY was associated with higher odds of high AIP risk (OR = 1.282, 95% CI: 1.186–1.387, *P* < 0.001), corresponding to a 28.2% increase in odds.

**Table 2:** Multivariable linear regression analysis of the association between HCY and AIP.

Model	Regression coefficient (*β*)	Standard error (SE)	Standardized coefficient (Beta)	*P*-value	Adjusted *R*^2^
Model 1	0.0131	0.0014	0.2944	<0.001	0.0857
Model 2	0.0124	0.0015	0.2769	<0.001	0.0891
Model 3	0.0113	0.0014	0.2526	<0.001	0.2572

HCY, homocysteine; AIP, atherogenic index of plasma; BMI, body mass index; SBP, systolic blood pressure; FBG, fasting blood glucose. Model 1 was unadjusted. Model 2 was adjusted for age and sex. Model 3 was further adjusted for BMI, SBP, FBG, smoking history, alcohol consumption history, and histories of hypertension, diabetes, and hyperlipidemia.

**Table 3:** Logistic regression analysis of the association between HCY and high AIP risk.

Model	Odds ratio (OR)	95% confidence interval (*CI*)	*P*-value
Model 1	1.284	1.198–1.377	<0.001
Model 2	1.268	1.179–1.364	<0.001
Model 3	1.282	1.186–1.387	<0.001

HCY, homocysteine; AIP, atherogenic index of plasma; OR, odds ratio; CI, confidence interval. Model definitions are identical to those used in Table 2.

To clarify which component of AIP was most closely related to HCY, AIP was further decomposed into log10(triglycerides) and log10(high-density lipoprotein cholesterol), which were analyzed separately as dependent variables. In Model 3, adjusted for age, sex, BMI, SBP, FBG, smoking and drinking history, and histories of hypertension, diabetes, and hyperlipidemia, each 1 micromol/L increase in HCY was associated with a 0.0085 increase in log10(triglycerides) (95% CI: 0.0059–0.0111, *P* < 0.001) and a 0.0026 decrease in log10(high-density lipoprotein cholesterol) (95% CI: −0.0043 to −0.0009, *P* = 0.003). These results indicate that higher HCY was independently related to both higher triglyceride levels and lower high-density lipoprotein cholesterol levels, thereby contributing jointly to higher AIP.

### 3.3 Mediation analysis

**Figure 4:**
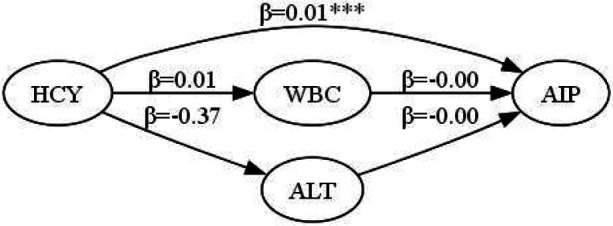
Path diagram used for the mediation analysis.

We next examined whether inflammation, represented by WBC, or liver function, represented by ALT, statistically mediated the association between HCY and AIP. The mediation model is illustrated in Figure 4, and the detailed estimates are provided in Table 4. Both the total effect of HCY on AIP (beta = 0.0113, *P* < 0.001) and the direct effect (beta = 0.0112, *P* < 0.001) were statistically significant. However, the indirect pathway through WBC was close to zero (effect value < 0.001, bootstrap 95% CI: −0.0002 to 0.0001), and the indirect pathway through ALT was similarly negligible (effect value < 0.001, bootstrap 95% CI: −0.0001 to 0.0003). Because both bootstrap confidence intervals included zero, neither indirect effect reached statistical significance. Thus, in this dataset, the HCY-AIP association appeared to be driven mainly by a direct pathway rather than by the selected inflammatory or liver-function markers.

**Table 4:** Mediation analysis of WBC and ALT in the association between HCY and AIP.

Path	Effect size	Bootstrap SE	95% bootstrap CI	*P*-value
Total Effect	0.0113	0.0014	(0.0085, 0.014)	<0.001
Direct Effect	0.0112	0.0014	(0.0085, 0.014)	<0.001
Indirect Effect (WBC)	<0.001	0.0001	(−0.0002, 0.0001)	0.732
Indirect Effect (ALT)	<0.001	0.0001	(−0.0001, 0.0003)	0.568

HCY, homocysteine; AIP, atherogenic index of plasma; WBC, white blood cell count; ALT, alanine aminotransferase; Boot SE, bootstrap standard error; CI, confidence interval. Bootstrap resampling was performed 5,000 times, and bias-corrected and accelerated (BCa) confidence intervals were calculated. *P* values were derived from the bootstrap distribution.

### 3.4 Subgroup analysis

**Figure 5:**
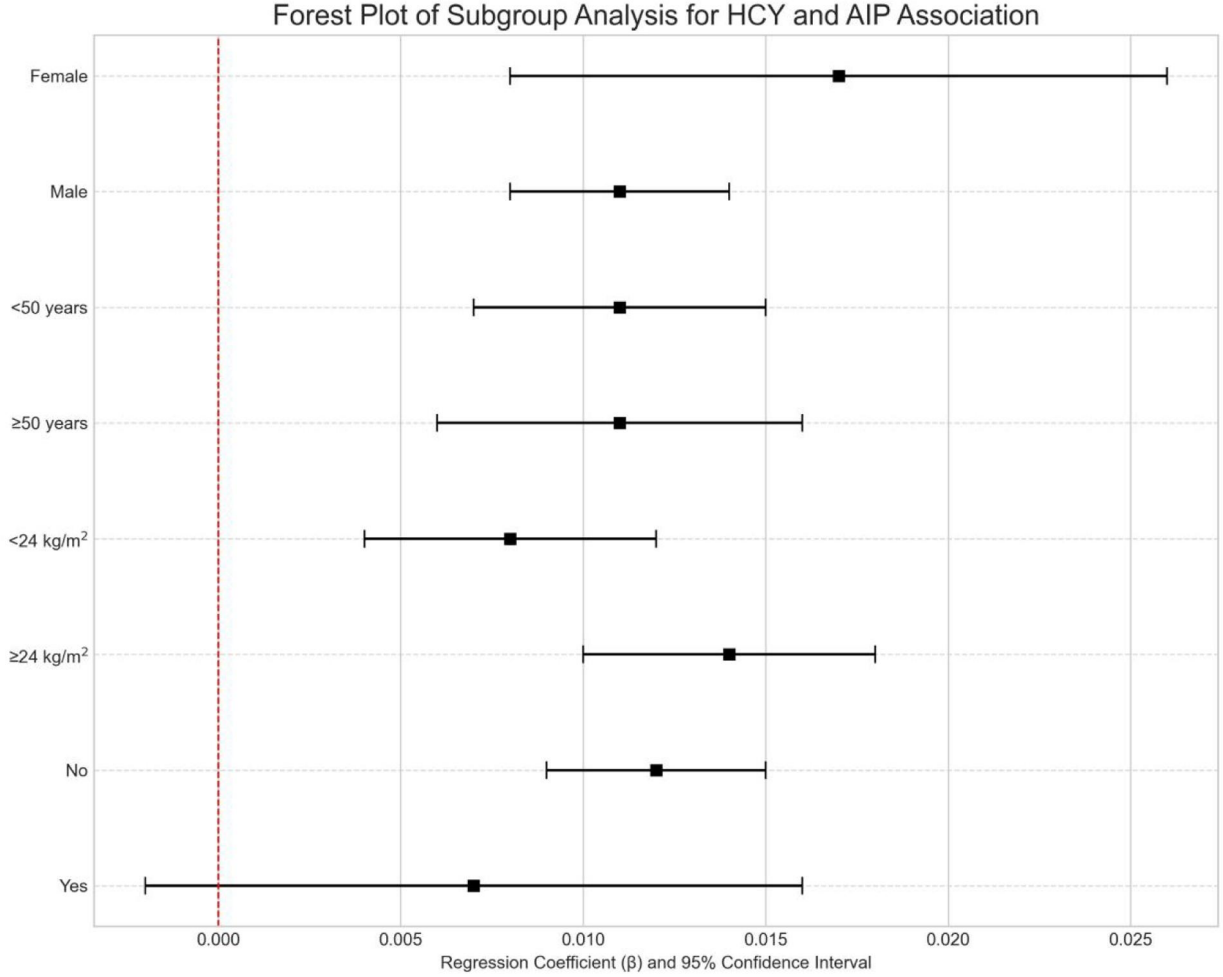
Forest plot of subgroup analyses for the association between HCY and AIP. HCY, homocysteine; AIP, atherogenic index of plasma; BMI, body mass index; CI, confidence interval.

Subgroup analyses were performed to evaluate whether the HCY-AIP association was consistent across clinically relevant strata (Figure 5 and Table 5). The positive association remained statistically significant in most subgroups, including both sexes, both age categories, and both BMI categories (all *P* < 0.05). A significant interaction was observed for BMI (*P* for interaction = 0.006): the association was stronger among participants with BMI ≥ 24 kg/m2 (beta = 0.014) than among those with BMI < 24 kg/m2 (beta = 0.008). By contrast, the association did not reach statistical significance in the hypertension subgroup (*P* = 0.102), and no significant interactions were detected for sex, age, or hypertension status.

**Table 5:** Subgroup analysis of the association between plasma homocysteine (HCY) and the atherogenic index of plasma (AIP).

Subgroup	N	Regression Coefficient (*β*)	95% *CI*	*P*-value	*P* for interaction
Sex					0.363
Female	85	0.017	(0.007, 0.026)	<0.01	
Male	802	0.011	(0.008, 0.014)	<0.001	
Age (years)					0.85
<50	603	0.011	(0.008, 0.015)	<0.001	
≥50	284	0.011	(0.006, 0.016)	<0.001	
BMI (kg/m^2^)					0.006
<24	446	0.008	(0.004, 0.012)	<0.001	
≥24	441	0.014	(0.011, 0.018)	<0.001	
Hypertension					0.232
No	776	0.012	(0.009, 0.015)	<0.001	
Yes	111	0.007	(−0.001, 0.016)	0.102	

## 4 Discussion

This cross-sectional study provides additional evidence that plasma homocysteine (HCY) has a close connection with the atherogenic index of plasma (AIP) in a general population. The main conclusions can be stated as follows: firstly, higher HCY concentrations are related to a worse cardiometabolic status; secondly, HCY shows a significant, dose-dependent and constant positive association with AIP after adjusting for major demographic and clinical factors; thirdly, an increase in HCY is connected with both higher AIP values and a greater possibility of being categorized into the high AIP group; fourthly, the pre-specified mediation analyses did not reveal any statistically significant indirect effects through white blood cell count (WBC) or alanine aminotransferase (ALT); fifthly, the HCY-AIP relationship was observed in most subgroups, but it was particularly strong among the overweight or obese individuals (BMI ≥ 24 kg/m^2^). In conclusion, these results suggest that the combination of high HCY and excessive body weight may represent a subgroup with a more atherogenic lipid profile.

The positive correlation between HCY and AIP is in agreement with the previous studies suggesting that hyperhomocysteinemia is associated with atherosclerotic risk and abnormal lipid metabolism (14, 15). HCY is believed to cause endothelial damage, smooth muscle cell proliferation, oxidative stress and metabolic disturbance, which may participate in the atherosclerotic remodeling (16, 17). In our research, the association still remained significant after controlling for age, sex, body mass index, blood pressure, fasting blood glucose, smoking, drinking habits and history of hypertension, diabetes and hyperlipidemia (*β* = 0.0113, *P* < 0.001). This result implies that the relationship between HCY and AIP is not solely due to the common cardiometabolic risk factors. The progressive rise of AIP in different quartiles of HCY further confirms a dose-response relationship and enhances the biological feasibility of the association. Moreover, the component analysis indicates that an increase in HCY is connected with both higher triglyceride levels and lower high-density lipoprotein cholesterol levels, showing that the change in AIP reflects concurrent alterations in both lipid components instead of a single-sided change in their ratio.

The outcomes of the mediation should be evaluated carefully. Neither WBC, which is a general indicator of systemic inflammation, nor ALT, functioning as a simple marker of hepatic function, have a great effect on the relation between HCY and AIP (Figure 4, Table 4). This finding does not deny the possibility that an inflammatory process or hepatic metabolism may be important pathways; rather, it suggests that these two common clinical tests may not be specific enough to reveal the true mechanisms in this situation. Some previous studies indicate that HCY may promote atherosclerosis through inflammatory stimulation or disturbance of hepatic fat metabolism (18–20). However, our results are more consistent with a direct relationship between HCY and AIP (21). Some possible direct mechanisms include endoplasmic reticulum stress, impairment of nitric oxide (NO) signal, oxidative damage to endothelium and other vascular toxic effects of HCY (22–24). The WBC can be affected by short-term infections, stress, smoking and metabolic inflammation, whereas ALT is influenced by fatty liver diseases, alcohol intake, drug use and muscle-related factors (25, 26). Further research using high-sensitivity C-reactive protein, interleukin-6, more detailed hepatic metabolic parameters, oxidative stress markers and omics-based indicators may be more suitable for studying indirect pathways (27–30). Additionally, the recent comprehensive review on the connection between AIP and coronary artery disease, adverse cardiovascular events and type 2 diabetes provides a broader clinical background for comprehending the relevance of the HCY-AIP relationship in assessing the cardiometabolic risks (31).

The modifying effect of BMI was one of the most clinically relevant observations in this study. The association between HCY and AIP was substantially stronger in participants with BMI ≥ 24 kg/m^2^ than in those with BMI < 24 kg/m^2^ (interaction *P* = 0.006). This pattern suggests that excess adiposity may amplify the atherogenic lipid signature associated with HCY. Prior work has shown that HCY is related to insulin resistance and may be elevated in obese individuals with hyperinsulinemia (32, 33). Obesity is also characterized by low-grade inflammation, adipose-tissue dysfunction, insulin resistance, and oxidative stress, creating a vascular and metabolic environment that may heighten susceptibility to HCY-related injury (34, 35). In this context, HCY may act on a background of endothelial dysfunction and lipid-metabolic impairment, thereby producing a stronger association with AIP (36). Adipokines such as leptin and adiponectin may also interact with one-carbon metabolism and lipid homeostasis, further linking obesity-related metabolic dysfunction to AIP elevation (37, 38). Clinically, these findings suggest that HCY should not be interpreted in isolation. In individuals with overweight or obesity, simultaneous attention to weight control, insulin sensitivity, and HCY-related nutritional factors may be more informative than evaluation of any single marker alone (39).

The hypertension subgroup analysis also deserves careful interpretation. The HCY-AIP association did not reach statistical significance among participants with hypertension (*β* = 0.007, *P* = 0.102), but the interaction by hypertension status was not significant (*P* for interaction = 0.232). Therefore, the result should not be taken as evidence that the association is absent in hypertensive individuals. Only 111 participants had hypertension, and this limited subgroup size reduced statistical precision, as reflected by the wide confidence interval (95% CI: −0.001 to 0.016). In addition, hypertension-related vascular injury, disease duration, and medication exposure may have obscured the incremental contribution of HCY to AIP. Antihypertensive agents can influence HCY metabolism in different directions; for example, some diuretics have been linked to higher HCY concentrations, whereas beta-blockers have been reported to lower HCY in some settings (40, 41). These competing effects may dilute the observed relationship. Prior studies have often focused on H-type hypertension, defined as hypertension with hyperhomocysteinemia, and have reported stronger links with atherosclerotic progression (42, 43). The present study did not specifically recruit an H-type hypertension cohort, which may partly explain the weaker subgroup signal. Larger studies with detailed blood-pressure phenotyping, medication information, and repeated HCY measurement are needed to clarify whether hypertension modifies the HCY-AIP relationship. For example, in a high-risk stroke population in Hainan Province, China, coexisting hypertension and hyperhomocysteinemia was associated with increased carotid intima-media thickness, with evidence of a more-than-additive interaction (44). In addition, data from CHARLS showed that AIP was associated with hypertension, diabetes, and their comorbidity in middle-aged and older Chinese adults (45).

From a lipid-risk perspective, AIP offers information that is not fully captured by conventional single lipid parameters. Although elevated HCY has been associated with hypertriglyceridemia and reduced high-density lipoprotein cholesterol (HDL-C), AIP integrates both abnormalities into a logarithmically transformed triglyceride-to-HDL-C ratio. This composite structure is clinically useful because it reflects the balance between triglyceride-rich lipoprotein burden and HDL-C-related protective capacity. AIP is also closely related to small, dense low-density lipoprotein (sdLDL), a highly atherogenic particle subtype that is rarely measured in routine screening. The independent HCY-AIP association observed here therefore suggests that HCY may be linked to a broader atherogenic lipid phenotype involving triglyceride enrichment, HDL-C depletion, and possibly sdLDL formation. Because AIP can be calculated from routine lipid measurements, it may be especially useful in occupational health examinations and primary-care risk screening. When HCY is assessed at the same time, combined interpretation of HCY and AIP may help identify individuals whose lipid-related atherogenic burden is underestimated by standard markers alone.

The magnitude of the association also warrants interpretation in clinically meaningful terms. Although the adjusted regression coefficient for HCY may appear small (*β* = 0.0113), AIP is a log10-transformed ratio; therefore, even modest absolute changes may correspond to meaningful proportional differences in the triglyceride-to-HDL-C balance. In this cohort, the median HCY level increased from 7.3 *μ*mol/L in the first quartile to 12.2 *μ*mol/L in the fourth quartile, an approximate difference of 5 *μ*mol/L. Applying the fully adjusted coefficient, this difference corresponds to an estimated AIP increase of about 0.0565, which is roughly equivalent to a 14% increase in the triglyceride-to-HDL-C ratio (10^0.0565^ ≈ 1.14). The logistic model provides a complementary interpretation: each 1 *μ*mol/L increase in HCY was associated with 28.2% higher odds of high AIP (OR = 1.282, 95% CI: 1.186–1.387). These calculations should not be interpreted causally because of the cross-sectional design, but they illustrate that population-level differences in HCY may translate into nontrivial differences in lipid-related atherogenic risk.

This study has several analytical strengths. Rather than relying on a single model, we evaluated the HCY-AIP relationship through correlation analysis, multivariable linear regression, quartile-based trend assessment, logistic regression for high AIP risk, component analysis of AIP, mediation analysis, and subgroup interaction testing. This layered approach allowed us to examine consistency, independence from confounding, dose-response behavior, risk classification, potential mechanisms, and population heterogeneity within the same dataset. The use of routine health-examination variables also increases the practical relevance of the findings, because both HCY and AIP can be obtained or calculated in many clinical screening settings without complex testing.

Overall, the findings point to a direct and BMI-sensitive relationship between HCY and AIP. The stronger association in participants with overweight or obesity indicates that adiposity may intensify the lipid-related expression of HCY-associated risk. At the same time, the negative mediation results for WBC and ALT help refine the mechanistic hypothesis by suggesting that routine inflammatory and liver-function markers do not adequately explain the association. Future studies should therefore move beyond broad markers and incorporate prospective follow-up, repeated biomarker assessment, more specific inflammatory and hepatic metabolic indicators, genetic information related to one-carbon metabolism, and measures of oxidative stress, endothelial function, adipokine signaling, and gut microbiota-derived metabolites. Such work will be important for determining whether HCY is merely a marker of risk or a modifiable contributor to the atherogenic lipid phenotype reflected by AIP.

## 5 Limitations

This study has several limitations. First, the cross-sectional design precludes causal inference and prevents determination of temporal sequence. Reverse causation cannot be excluded. For example, lipid abnormalities reflected by a higher AIP could theoretically influence HCY metabolism through lipotoxicity-related liver injury or alterations in the transsulfuration pathway (46, 47), rather than HCY acting unidirectionally on AIP. Second, the study sample had a high proportion of men. Although sex was included as a covariate and subgroup analyses were performed, the generalizability of the findings should be confirmed in cohorts with a more balanced sex distribution. Third, HCY and AIP were each measured only once; therefore, long-term variability, regression dilution, and dynamic changes over time could not be assessed. Fourth, the mediation analysis relied on WBC and ALT, which are imperfect markers. WBC is a nonspecific indicator of systemic inflammation and cannot distinguish infection-related, metabolic, or other inflammatory sources. ALT is influenced by non-alcoholic fatty liver disease, alcohol intake, medication exposure, muscle injury, and other conditions and is therefore not a specific measure of hepatic metabolic function. Although overt acute infection was screened during physical examination, mild or subclinical inflammation could not be completely excluded and may have weakened the usefulness of WBC as a marker of chronic inflammation. More importantly, mediation analysis based on cross-sectional data cannot establish the temporal ordering of exposure, mediator, and outcome. The estimated indirect effects should therefore be interpreted as statistical decompositions rather than causal mediation. Finally, residual confounding remains possible despite adjustment for prespecified covariates. Unmeasured factors such as dietary folate and B-vitamin intake, renal function, detailed physical activity, chronic stress, alcohol dose, medication use, and genetic variants such as MTHFR C677T may influence both HCY and AIP. The negative mediation findings should therefore be considered hypothesis-generating and should be revisited using prospective designs, Mendelian randomization, repeated biomarker measurements, and more specific inflammatory or hepatic markers, such as high-sensitivity C-reactive protein, interleukin-6, gamma-glutamyl transferase, or imaging-based assessment of hepatic steatosis.

## 6 Conclusion

In summary, plasma HCY was independently and positively associated with AIP in this health examination population, and the association was stronger among participants who were overweight or obese. These findings suggest that HCY may mark an adverse atherogenic lipid phenotype beyond conventional risk factors. No significant mediation by WBC or ALT was observed, indicating that the association was not explained by the selected systemic inflammation or liver function markers in this dataset. Because the study was cross-sectional, the results should be interpreted as hypothesis-generating rather than as causal evidence or direct clinical guidance. If confirmed in prospective and interventional studies, combined evaluation of HCY, AIP, and BMI may help refine atherosclerotic risk stratification, particularly among overweight or obese adults.

## Data Availability

The original contributions presented in the study are included in the article/Supplementary Material, further inquiries can be directed to the corresponding author/s.
